# Correction: Free long-chain fatty acids trigger early postembryonic development in starved *Caenorhabditis elegans* by suppressing mTORC1

**DOI:** 10.1371/journal.pbio.3004083

**Published:** 2026-10-27

**Authors:** Meiyu Ruan, Fan Xu, Na Li, Jing Yu, Fukang Teng, Jiawei Tang, Cheng Huang, Huanhu Zhu

There is an error in the Funding statement. The correct Funding statement is as follows: This work was supported by the National Natural Science Foundation of China (32170837)(HZ), the National Key R&D Program of China, 2019YFA0802804[HZ],2021YFA0804700[HZ]), Double First-Class Initiative Fund of ShanghaiTech University (SYLDX0162022)(HZ), Science& Technology Commission of the Shanghai Municipality (16PJ1407400)(HZ), the Recruitment Program of Global Experts of China (Youth)(HZ), and ShanghaiTech Startup program (HZ). Funders did not play any role in the study design, data collection and analysis, decision to publish, or preparation of the manuscript.
