# Supplementary material for: Free long-chain fatty acids trigger early postembryonic development in starved Caenorhabditis elegans by suppressing mTORC1
Source: PLoS Biol. 2024 Oct 22;22(10):e3002841. doi: 10.1371/journal.pbio.3002841 (PMC11530034; doi:10.1371/journal.pbio.3002841)
Supplement: S1 Fig — (A) Fluorescent microscopic pictures showing the maturation of AWC sensory neurons marked by STR-2::GFP. Mature AWC neurons were indicated by arrowheads. Related to Fig 1D. (B) A statistical bar graph showing the percentage of animals with mature AWC neurons under various nutrient supplementations. Mean ± SEM. Ordinary one-way ANOVA. Related to Fig 1E. (C) A picture showing the chemotaxis assay to measure whether animals could be attracted to butanone on NGM plates. Related to Fig 1F. (D, E) A text description in Knight and colleagues [29] showing the seam cell development of C. elegans larvae at different time stages under the fed condition (D). Fluorescent microscopic pictures showing the seam cells (marked by AJM-1::GFP, a marker for adherens junctions) in palmitic acid (PA) fed L1 animals. Divided seam cells were indicated by asterisks (E). Related to Fig 1G. (F) Fluorescent microscopic pictures showing the seam cell marker SCM (nuclear signal). The nuclei of seam cells were indicated by arrowheads. Divided seam cells were indicated by asterisks. (G) A cartoon picture modified from Ou, G. and Vale, R. D. [33] illustrating the Q cell migration. Related to Fig 1I. (H) MEC-4::GFP expressed in 6 gentle touch-sensing neurons including AVM and PVM (third division of Q cell), which could not be observed in either solvent or PA-fed groups. (I) Fluorescent microscopic pictures showing M cell division marked by HLH-8::GFP with/out palmitic acid (PA) supplementation. None of M cells was divided in both conditions. The data underlying the graphs shown in the figure can be found in S1 Data. (PDF) [file pbio.3002841.s001.pdf]

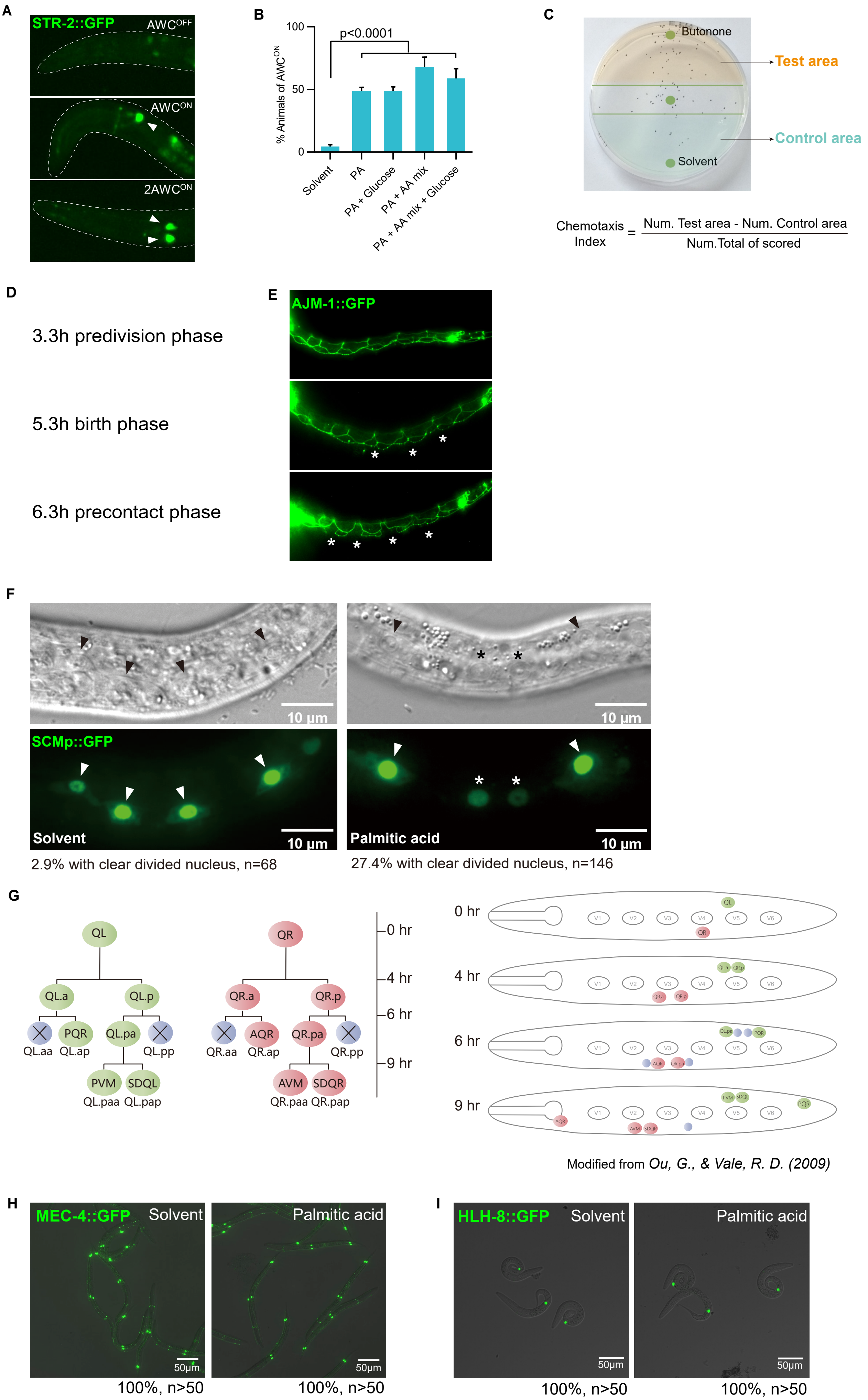

S1 Fig. Related to Fig 1. Palmitic acid promoted the development of arrested L1 *C. elegans* under starvation.
