# Supplementary figures and images for: Free long-chain fatty acids trigger early postembryonic development in starved Caenorhabditis elegans by suppressing mTORC1
Source: PLoS Biol. 2024 Oct 22;22(10):e3002841. doi: 10.1371/journal.pbio.3002841 (PMC11530034; doi:10.1371/journal.pbio.3002841)

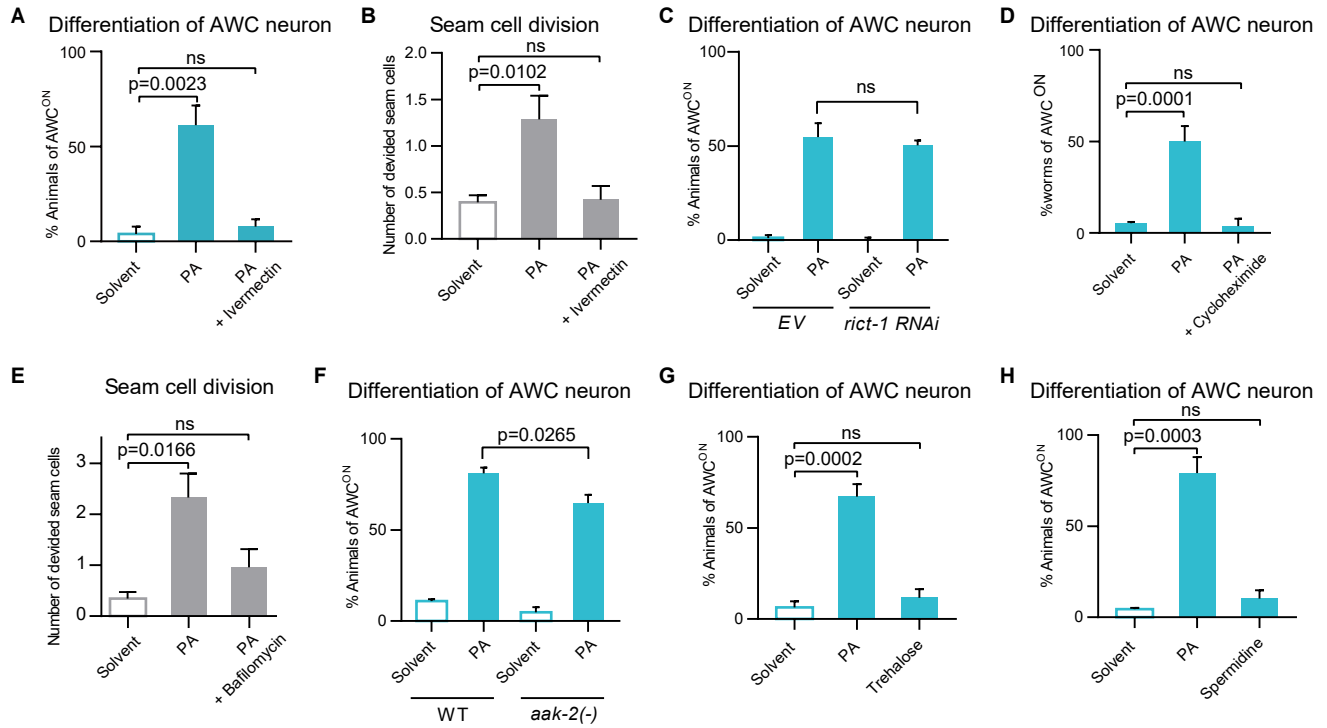

**S2 Fig. Related to Fig 2. Inactivation of mTORC1 was necessary and sufficient to mediate FEDUS.**

Supplement: S2 Fig — (A, B) A bar graph showing the percentage of animals with mature AWC neurons (A) and the average number of divided seam cells (B). Animals treated with anesthetics ivermectin (20 ng/ml) exhibited greatly decreased FEDUS. Related to Fig 2A. (C) WT or rict-1(-) animal, under solvent or palmitic acid (PA) supplementation, showed no difference in the AWC maturation. EV, empty vector. (D) A bar graph showing the percentage of animals with mature AWC neurons; 1.5 mM cycloheximide, a eukaryotic translation inhibitor, could suppress FEDUS. PA, palmitic acid. (E) The autophagy inhibitor bafilomycin (25 μg/ml) suppressed seam cell division in FEDUS. Related to Fig 2J. (F) Loss function of aak-2 (ok524) did not suppress FEDUS, as indicated by the percentage of animals with mature AWC neurons. (G, H) Autophagy activators, up to100 mM trehalose (G) or 10 mM spermidine (H) treatment did not activate AWC neuron maturation. All statistical data are represented as mean ± SEM. Ordinary one-way ANOVA. ns, not significant. The data underlying the graphs shown in the figure can be found in S1 Data. (PDF) [file pbio.3002841.s002.pdf]

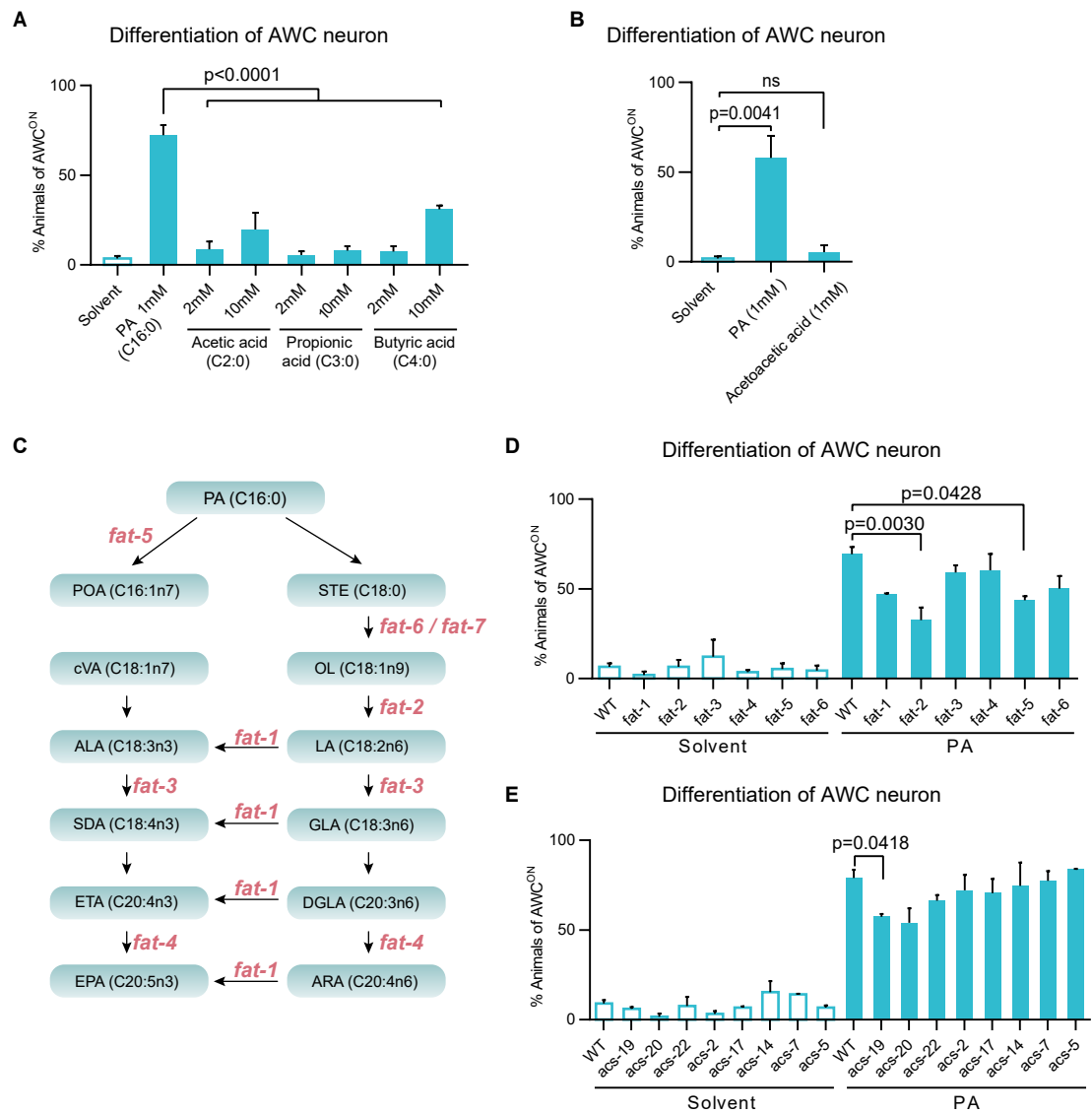

**S3 Fig. Related to Fig 3. Fatty acid catabolism and fatty-acylation were not required for FEDUS.**

Supplement: S3 Fig — (A, B) Bar graphs showing the percentage of animals with mature AWC neurons. Animals supplied with various concentrations of short chain fatty acids were tested (A). Supplementation of acetoacetate, a ketone body, could not initiate the AWC maturation (B). (C) A cartoon illustration of the long-chain fatty acid elongation pathway. PA, palmitic acid; POA, palmitoleic acid; STE, stearic acid; cVA, cis-vaccenic acid; OL, oleic acid; LA, linolenic acid; ALA, α-linolenic acid; GLA, γ-linolenic acid; SDA, stearidonic acid; DGLA, dihomo-γ-linolenic acid; ETA, eicosatetraenoic acid; ARA, arachidonic acid; EPA, eicosapentaenoic acid. (D, E) Bar graphs showing the percentage of animals with mature AWC neurons. (D) WT and multiple genetic mutants of long-chain fatty acids dehydrogenases [fat-1(ok2323), fat-2(wa17), fat-3(ok1126), fat-4(ok958), fat-5(tm420), fat-6(tm331)] in the PUFA biosynthetic pathway were tested. (E) WT and various mutants of Acyl-CoA synthase [acs-2(ok2457), acs-5(ok2668), acs-7(tm6781), acs-14(ok3391), acs-17(ok1562), acs-19(tm4853), acs-20(tm3232), acs-22(tm3236)] under solvent or palmitic acid (PA) supplement were tested. Among PA-supplement groups, only the P-values with significant differences between the groups were labeled. All statistical data are represented as mean ± SEM. Ordinary one-way ANOVA. ns, not significant. The data underlying the graphs shown in the figure can be found in S1 Data. (PDF) [file pbio.3002841.s003.pdf]

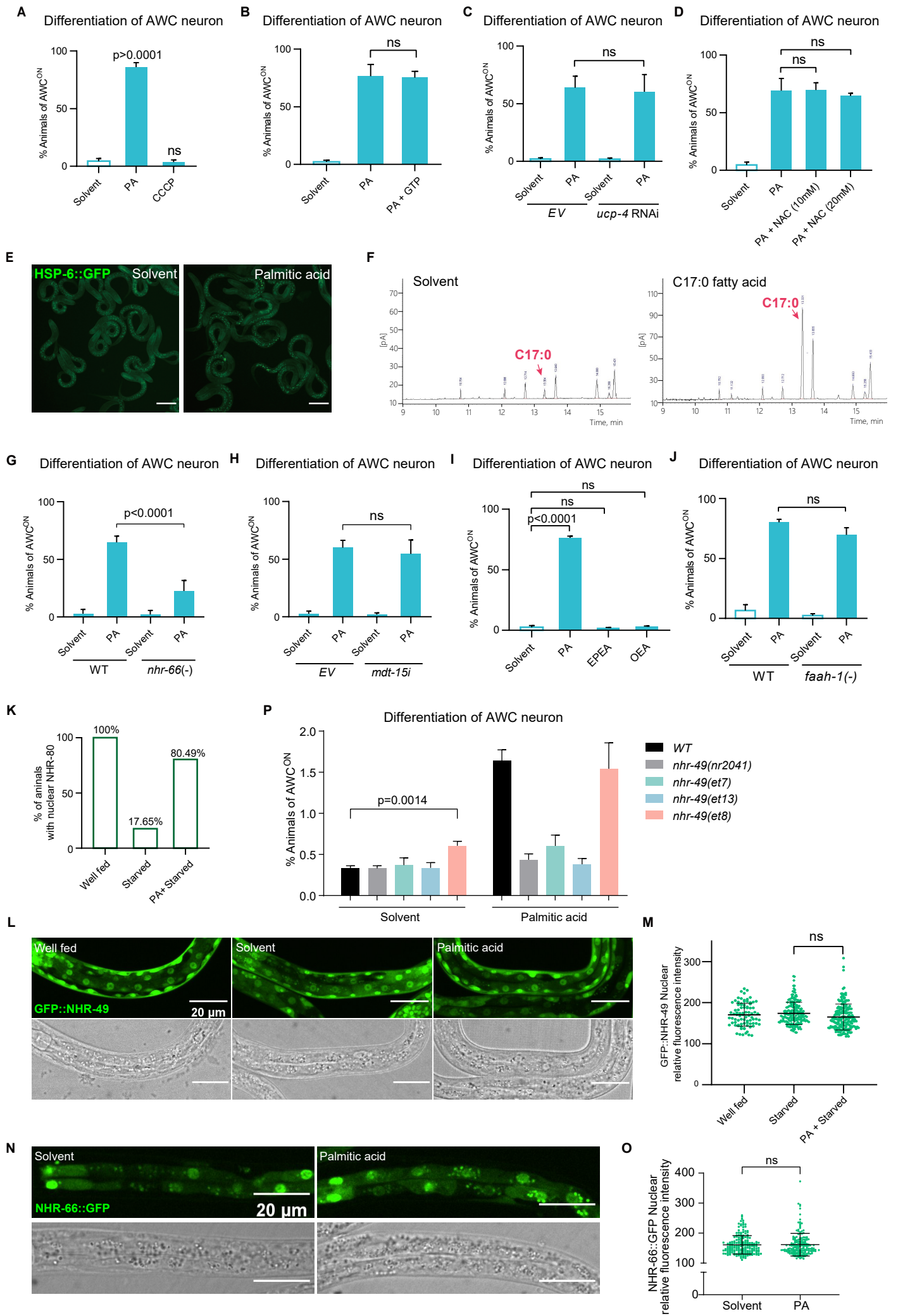

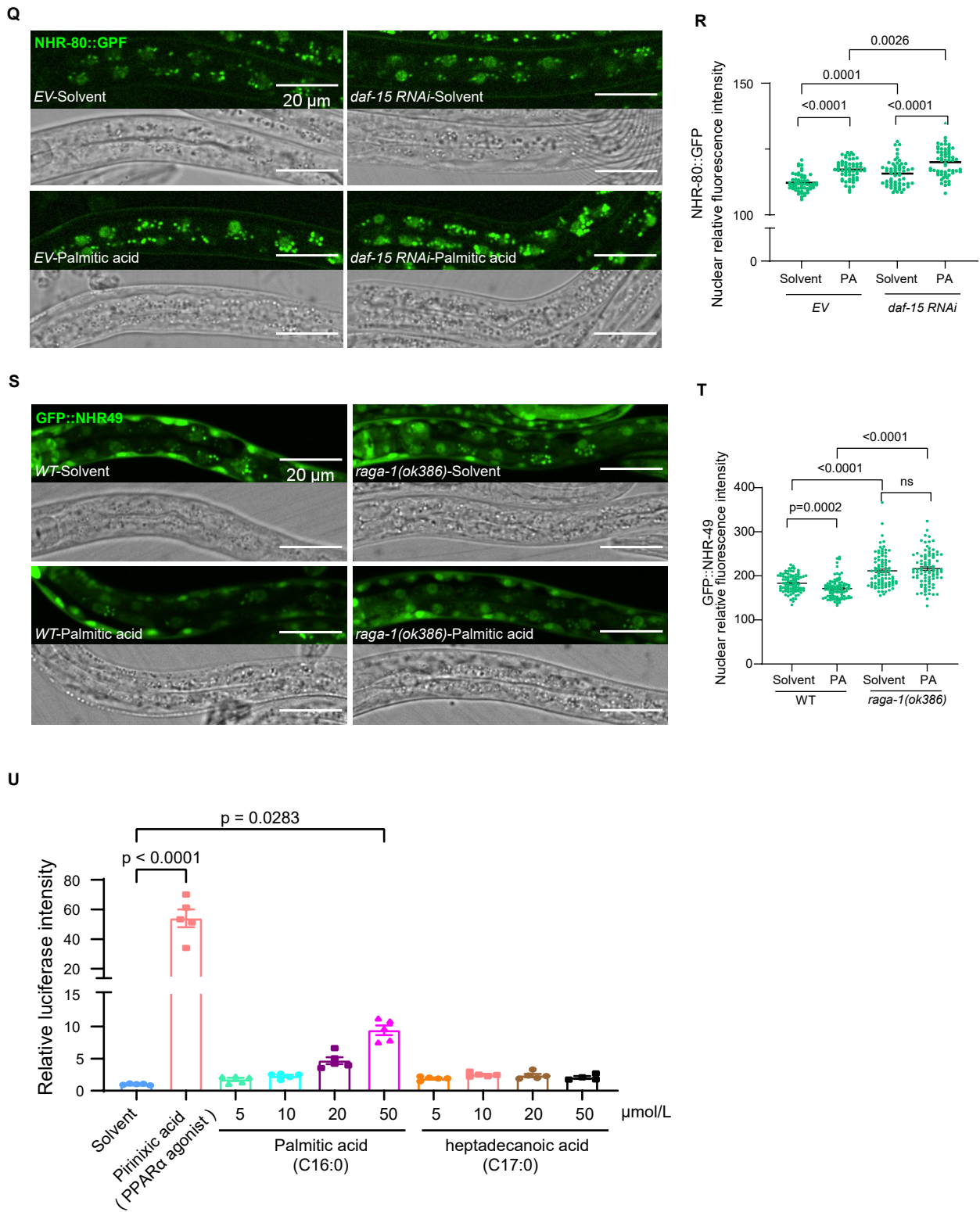

**S4 Fig. Related to Fig 4. NHR-49/80 mediated FEDUS via the peroxisomal activation.**

Supplement: S4 Fig — (A–D) Bar graphs showing the percentage of animals with mature AWC neurons. The mitochondrial respiratory chain decoupler CCCP (15 μm) did not initiate the maturation of AWC neurons. Supplementation of 1 mM GTP(B), RNAi of ucp-4 (C), or the ROS inhibitor NAC (D) could not inhibit the maturation of AWC neurons. EV, empty vector. (E) Representative fluorescent microscopic images showing the ROS level indicated by HSP-6::GFP under solvent or palmitic acid supplementation. There was no significant difference between these 2 groups. (F) Gas chromatography plot showing the fatty acids profile of L1 animals. Dietary supplementation of heptadecanoic acid (C17:0, red arrows) was indeed absorbed by C. elegans. (G) Bar graphs showing the loss function of nhr-66 (ok940) facilitated AWC neuron maturation in FEDUS. (H) Bar graphs showing the RNAi of mdt-15 did not affect FEDUS. EV, empty vector. (I) Supplementation of EPEA (eicosapentaenoyl ethanolamide) or OEA (oleylethanolamide), 2 different NAEs, had no effect on the maturation of AWC neurons. (J) Mutation of faah-1 (tm5011) had no effect on the maturation of AWC neurons. (K) A bar graph showing the percentage of animals with nucleus-localized NHR-80::GFP. Palmitic acid supplementation dramatically increased the nuclear localization of NHR-80::GFP under starvation. Related to Fig 4D. (L) Representative fluorescent microscopic images showing the subcellular localization of GFP::NHR-49 under various nutrient conditions (upper panel). Related bright field microscopic pictures were also shown (bottom panel). (M) A statistical bar graph showing the percentage of animals with nucleus-localized GFP::NHR-49. For qualification, the fluorescence intensity of 6 intestinal nuclei on one side of each animal were counted (at least 30 animal per condition for each biological replicate). There was no statistical difference between starvation and palmitic acid (PA) supplementation groups. (N) Representative fluorescent microscopic images showi [file pbio.3002841.s004.pdf]

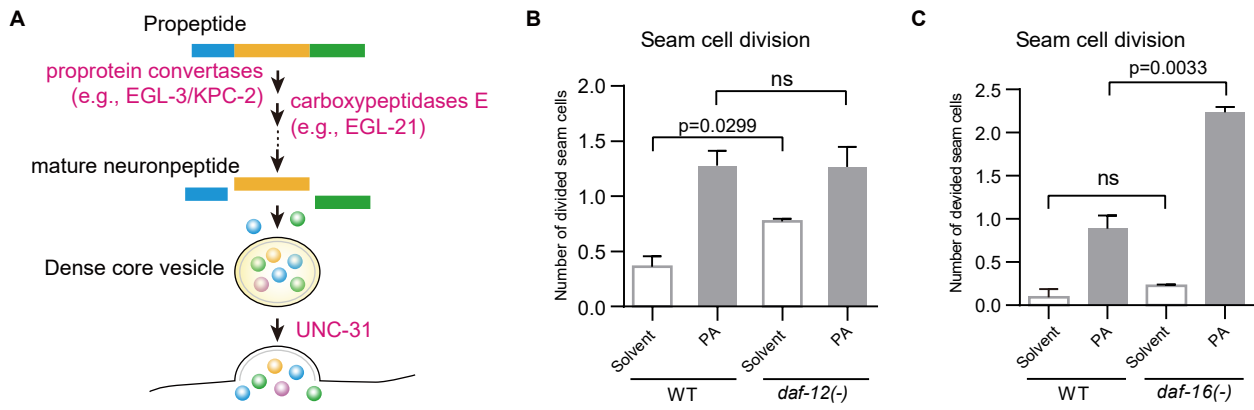

**S7 Fig. Related to Fig 5. Ciliated sensory neurons and the insulin-like pathway mediated FEDUS.**

Supplement: S7 Fig — (A) A chart showing the neuropeptide processing and secretion pathway. Related to Fig 5B–5D. (B, C) Bar graphs showing the average number of divided seam cells. Significant seam cell division was observed in daf-12 mutant (rh61rh412). All statistical data are represented as mean ± SEM. Ordinary one-way ANOVA. ns, not significant. The data underlying the graphs shown in the figure can be found in S1 Data. (PDF) [file pbio.3002841.s007.pdf]
