# Supplementary material for: Free long-chain fatty acids trigger early postembryonic development in starved Caenorhabditis elegans by suppressing mTORC1
Source: PLoS Biol. 2024 Oct 22;22(10):e3002841. doi: 10.1371/journal.pbio.3002841 (PMC11530034; doi:10.1371/journal.pbio.3002841)
Supplement: S5 Fig — (A) Fluorescent microscopic pictures showing the intestine mitochondria morphology indicated by GES-1::GFP(mit) under treatment of indicated RNAi. Tubular mitochondria morphology was observed in palmitic acid treated worms. RNAi of eat-3 disrupted the tubular shape and exhibited totally fragment shape. Drp-1 RNAi worms exhibited disrupted mitochondria structure, tending to form blebs and somewhat tubular shape, compared to the completely starved animals. EV, empty vector. (B) Bar graphs showing the percentage of animals with mature AWC neurons. RNAi of drp-1 and eat-3 could not inhibit the maturation of AWC neurons. (C) A bar graphs showing the loss function of unc-116 (e2310) inhibited mature AWC neurons. Related to Fig 4K. (D) A bar graph showing tissue specific rescue of UNC-116 in unc-116 (e2310) mutants. Pges-1 and Prgef-1 were specific promoters in intestine and neuron tissue, respectively. (E–H) Bar graphs showing the percentage of animals with mature AWC neurons. Supplementation of 10 mM mevalonic acid (E) or 1 mM coenzyme Q9 (F) did not initiate the maturation of AWC neurons; 1 mM lovastatin (G), an HMG-CoA reductase inhibitor, or (H) a daf-22 loss-of-function mutant (ok693), could not suppress the FEDUS. The data underlying the graphs shown in the figure can be found in S1 Data. (PDF) [file pbio.3002841.s005.pdf]

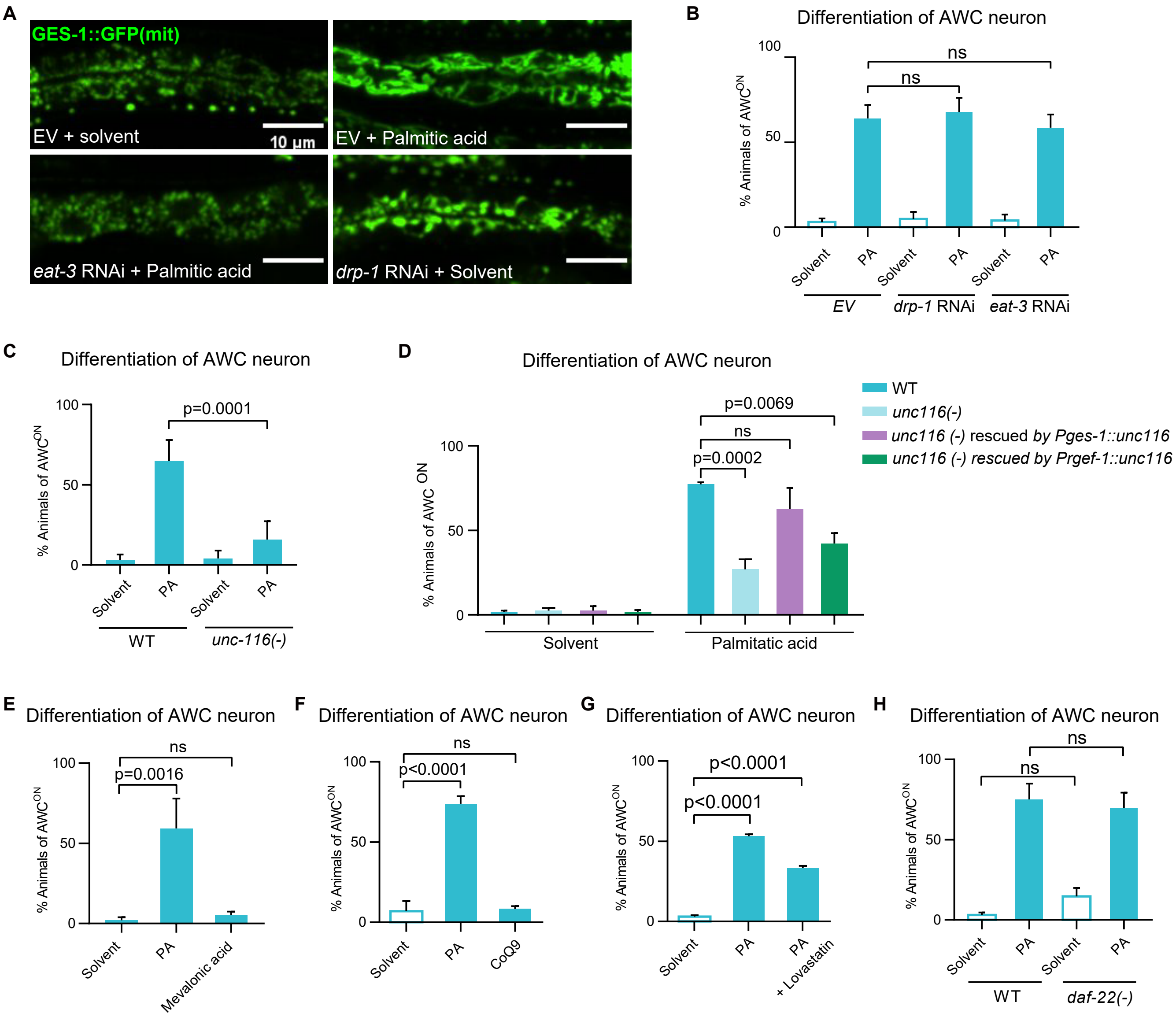

S5 Fig. Related to Fig 4. A secretive hormone derived from apical intestine-positioned peroxisomes mediated FEDUS.
