# Supplementary material for: Free long-chain fatty acids trigger early postembryonic development in starved Caenorhabditis elegans by suppressing mTORC1
Source: PLoS Biol. 2024 Oct 22;22(10):e3002841. doi: 10.1371/journal.pbio.3002841 (PMC11530034; doi:10.1371/journal.pbio.3002841)
Supplement: S6 Fig — (A, B) NHR-49 interaction proteins were analyzed using GeneMANIA (A). The heatmap (B) showing expression of the 20 highest-ranking selected genes under various nutrient conditions. (C) A heatmap showing the gene expression of L1 animals under various nutrient conditions. Related to Fig 1M. (D) A heatmap showing the peroxisomal β-oxidation related genes of L1 animals under various nutrient conditions. (E) A Venn diagram of up-regulated genes (compared to the solvent group) among 3 different expression gene sets (DEGs) shown by 3 colors. A total of 1,124 genes were up-regulated in the solvent (DMSO) vs. palmitic acid (PA) group. (F) A KEGG pathway analysis chart of the 1,124 genes depicted in the Venn diagram (E). Peroxisome-related genes are highly enriched. (G) Expression changes of genes in the peroxisome group (indicated in red in S5B) were listed. The data underlying the graphs shown in the figure can be found in S1 Data. (PDF) [file pbio.3002841.s006.pdf]

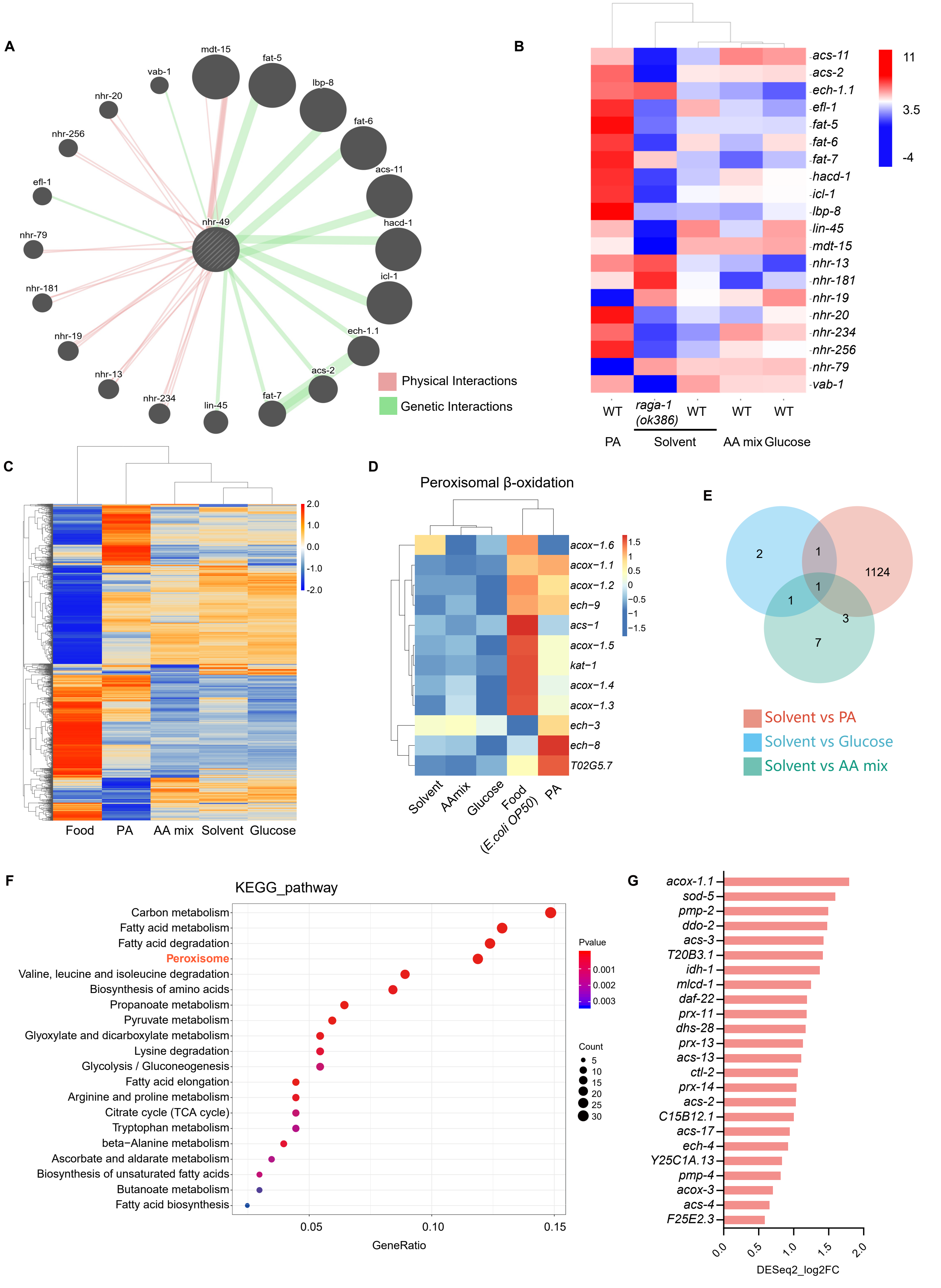


**S6. Related to Figs 1 and 4. Transcriptional analyses of *C. elegans* under various genetic and nutritional conditions.**
