# Supplementary material for: Free long-chain fatty acids trigger early postembryonic development in starved Caenorhabditis elegans by suppressing mTORC1
Source: PLoS Biol. 2024 Oct 22;22(10):e3002841. doi: 10.1371/journal.pbio.3002841 (PMC11530034; doi:10.1371/journal.pbio.3002841)
Supplement: S1 Table — (DOCX) [file pbio.3002841.s008.docx]

|  | *Table S1 C. elegans strains used in this paper.* |  |
| --- | --- | --- |
| **Strain name** | **Genotype** | **Source of strain** |
| Transgenic fluorescence strains: | | |
| SU93 | *jcIs1 [ajm-1::GFP + unc-29(+) + rol-6(su1006)]* | CGC |
| CX3695 | *kyIs140 [str-2::GFP + lin-15(+)]* | CGC |
| SJ4100 | *hsp-6::GFP(zcIs13)* | CGC |
| SJ4143 | *zcIs17[ges-1::GFP(mit)].* | CGC |
| DA2123 | *adIs2122 [lgg-1p::GFP::lgg-1 + rol-6(su1006)].* | CGC |
| PD4667 | *ayIs6 [hlh-8::GFP+ dpy-20(+)].* | CGC |
| SK4005 | *zdIs5 [mec-4::GFP + lin-15(+)].* | CGC |
| RDV55 | *rdvIs1 [egl-17p::MyrimCherry::pie-1 30UTR + egl-17p::mig-10::YFP::unc-54 30UTR + egl-17p::mCherry-TEV-S::his-24 + rol-6 (su1006)]* | CGC |
|  | *scm* |  |
| HGA8001 | *nhr-80::gfp {lynEx1[(pJG01)nhr-80p::nhr-80::GFP + myo-2p::dsRed]* | CGC |
| ZHU402 | *zmnEx[(rpl28p::Histone::mcherry);sydIs031[pha-4p::pha-4::GFP-3*FLAG]]* | Zhu Huanhu Lab |
| ZHU505 | *N2 Is[Pdaf-22::gfp::daf-22] knock in* | Zhu Huanhu Lab |
| Mutant strains were crossed with *kyIs140* and/or *jcIs1:* | | |
| VC222 | *raga-1(ok386)* | CGC |
| VC20125 | *prx-11(gk959960)* | CGC |
| FF41 | *unc-116(e2310)* | CGC |
| RB859 | *daf-22(ok693)* | CGC |
| TM3425 | *epg-5(tm3425)* | CGC |
| CB1370 | *daf-2(e1370)* | CGC |
| AA18 | *daf-12(rh61rh412)* | CGC |
| SP1732 | *osm-6(m201)* | CGC |
| GR1307 | *daf-16(mgDf50)* | CGC |
| RB754 | *aak-2(ok524)* | CGC |
| CB928 | *unc-31(e928)* | CGC |
| TM4948 | *prx-5(tm4948)* | CGC |
| tm5011 | *faah-1 (tm5011)* | CGC |
| FF41 | *unc-116 (e2310)* | CGC |
| nr2090 | *egl-3(nr2090)* | CGC |
| KP2018 | *egl-21(n476)* | CGC |
| HZ1675 | *atg-7(bp422)* | CGC |
| HZ1684 | *atg-3(bp412)* | CGC |
| SOZ0875 | *fat-1(ok2323)* | CGC |
| SOZ0948 | *fat-2(wa17)* | CGC |
| SOZ0877 | *fat-3(ok1126)* | CGC |
| SOZ0878 | *fat-4(ok958)* | CGC |
| SOZ0879 | *fat-5(tm420)* | CGC |
| SOZ0601 | *fat-6(tm331)* | CGC |
| RB2452 | *acs-14(ok3391)* | CGC |
| RB1899 | *acs-2(ok2457)* | CGC |
| RB2015 | *acs-5(ok2668)* | CGC |
| tm6781 | *acs-7(tm6781)* | CGC |
| RB2452 | *acs-14(ok3391)* | CGC |
| RB1377 | *acs-17(ok1562)* | CGC |
| tm4853 | *acs-19(tm4853)* | CGC |
| tm3232 | *acs-20(tm3232)* | CGC |
| FXO3236 | *acs-22(tm3236)* | CGC |
| JT11067 | *xbx-1(ok279)* | CGC |
| CB1377 | *daf-6(e1377)* | CGC |
| STE68 | *nhr-49(nr2041)* | CGC |
| tm1011 | *nhr-80 (tm1011)* | CGC |
| STE69 | *nhr-66 (ok940)* | CGC |
| Transgenic strains originally used in this paper: | | |
|  | *kyIs140 Ex[rpl-28p::raga-1 + myo-2p::dsRed]* | This paper |
|  | *kyIs140 Ex[ges-1p::raga-1 + myo-2p::dsRed]* | This paper |
|  | *kyIs140 Ex[rgef-1p::raga-1 + myo-2p::dsRed]* | This paper |
|  | *kyIs140 Ex[dpy-7p::raga-1 + myo-2p::dsRed]* | This paper |
|  | *kyIs140 Ex[nhr-80p::nhr-80* *+ myo-2p::dsRed]* | This paper |
|  | *kyIs140 Ex[ges-1p::nhr-80 + myo-2p::dsRed]* | This paper |
|  | *kyIs140 Ex[rgef-1p::nhr-80 + myo-2p::dsRed]* | This paper |
|  | *kyIs140 Ex[dpy-7p::nhr-80 + myo-2p::dsRed]* | This paper |
|  | *kyIs140 Is[nhr-49p::GFP::nhr-49 + myo-2p::dsRed]* | This paper |
|  | *kyIs140 Ex[nhr-66p::nhr-66* *+ myo-2p::dsRed]* | This paper |
|  | *unc-116(e2310) ex[unc-116p::unc-116;myo-2p::gfp]* | This paper |
|  | *unc-116(e2310) ex[ges-1p::unc-116;myo-2p::gfp]* | This paper |
|  | *unc-116(e2310) ex[rgef-1p::unc-116;myo-2p::gfp]* | This paper |
|  |  |  |
