# Supplementary material for: Free long-chain fatty acids trigger early postembryonic development in starved Caenorhabditis elegans by suppressing mTORC1
Source: PLoS Biol. 2024 Oct 22;22(10):e3002841. doi: 10.1371/journal.pbio.3002841 (PMC11530034; doi:10.1371/journal.pbio.3002841)
Supplement: Attachment — Submitted filename: Point to point reponse letter.docx [file pbio.3002841.s013.docx]

Reviewers' comments

*Rev. 1:*

*General comments:*

*This manuscript describes a series of genetics-based experiments that led to the finding of a highly novel and significant mechanism underlying the regulatory role of a nutrient signal in the initiation of postembryonic development. The novelty of the study lies in both the initial finding of the role of long-chain fatty acid and the mechanism underlying such a role. The ability of one LCFA to initiate postembryonic development was a very surprising and intriguing finding that would impact our thinking about nutrient sensing in general. The finding that mTORC1 negatively regulates the LAFA-triggered initiation of development is incredibly interesting and provocative. The data on other aspects of the mechanism, including roles of two nuclear receptors, peroxisome-produced factors, and sensory neurons, make this quite a complete story. This study reminds us again that nutrient sensing mechanisms need to be explored and analyzed in true in vivo and physiological*

*function-relevant conditions. The authors did a good job in the introduction to explain the rationale of the hypothesis and approach they took in chasing a single nutrient as the growth signal. The experimental data are mostly solid and well represented. With some relatively minor modifications, publication in PLoS Biology is highly recommended.*

We really appreciate the positive response from Reviewer#1, as well as the insightful suggestions raised below. We followed those suggestions and substantially improved our manuscript through additional experiments, discussion and other modifications.

*Specific comments*

*1. The title of the paper may include information about the role of mTOC1 and nuclear receptors, both of which are among critically significant findings of this work. Adding the information will make the work more attractive to readers.*

This is a great suggestion. We modified the title to “Free long chain fatty acid solitarily launches early postembryonic development in *Caenorhabditis elegans* **by suppressing mTORC1** under starvation” according to reviewer#1’s suggestion. Given NHR-49/80 is only necessary but not sufficient to initiate the L1 development, we think it may be better not to include it in the title.

*2. Why would animals use LCFA, not AA or Carb, as the signal to trigger the development at a very early postembryonic stage? Is LCFA a better nutrient signal to represent the overall nutrient and metabolic status? The authors might add some discussion on this.*

This is a fantastic question. We thought there may be several reasons. First, for a living animal that uses its digestive organs to uptake nutrients, LCFA (compared to AA or carbohydrates) may be a more reliable metabolite to indicate the availability of food in nature. After all, LCFA is the major basic building block of the cell membrane and hardly comes from other sources, while amino acids or carbohydrates could derive from extracellularly secretive or degrative metabolites. Second, LCFA is also a basic nutrient directly absorbed by the intestine (the TAG in food has to be hydrolyzed to LCFA by several lipases in the digestive duct before it can be absorbed by enterocytes). On the contrary, most peptides were absorbed in the form of di/tri-peptide rather than free amino acids. Finally, the energy intensity of fatty acid is much higher than amino acid or glucose, which could be critical for the very early development. Therefore, animals may develop a LCFA sensing system in the early developmental stage to regulate the developmental fate during evolution. Interestingly, C. elegans seems to develop a mechanism to make sure only exogenous LCFA, rather than LCFAs derived from other metabolites (such as TAG hydrolysis or de novo fatty acid biosynthesis from amino acids or carbohydrates), could trigger the development under starvation. When the development was initiated, genes in TAG hydrolysis or de novo LCFA biosynthesis would be highly upregulated. Further study is necessary to understand how those regulations have been molecularly achieved.

We have added several sentences in the discussion accordingly.

*3. The negative role of TORC1 is truly surprising and interesting to me. The data supporting such a role is excellent. The mention of the positive role of TORC1 in promoting development and growth in a later stage is good, but the discussion may need some modification to make the difference clear to the readers. The positive role was supposed to be there in well-fed animals and deactivated in nutrient deprivation condition, which indicates the role of mTORC1 in promoting postembryonic development. It might be good for readers if the authors would mention the known downstream events in the known positive role described in the previous studies.*

This is a great question, we have added a few sentences in the discussion to distinguish the two roles of mTORC1 at the early development. We have also introduced known downstream pathways of mTORC1 under the well-fed condition in the discussion.

*4. The authors may need to do more to explain/discuss the downstream roles of mTORC1 in the pathway. I am very glad that autophagy was analyzed, and the authors provide solid data to indicate that autophagy activation was necessary but not sufficient for FEDUS. It seems logical that producing a limited amount of AA and other molecules from autophagy is needed for FEDUS, as cell divisions need at least some of the macronutrients under starvation. Citing a previous study that indicated a link between mTORC1 inhibition and PPARalpha activation in mammals, the authors seem to suggest that the main regulatory role of deactivating mTORC1 was to activate NHR-49/80 in the mechanism for FEDUS. Such a proposed relationship was shown in Figure 5G as a hypothetical interaction (dotted arrow), which was reasonable given the data presented about the roles of the NHR proteins. However, this work did not provide data to support the relationship. Is it technically feasible for the authors to check the NHR activity (nuclear localization, activity of know target genes, etc) under the condition when mTORC1 is artificially deactivated in starved L1 larvae to actually nail the connection? If not, the authors may modify the writing to clearly discuss the two above roles and possibly other roles of mTORC1 in the pathway.*

We really appreciate those sharp and insightful questions/suggestions raised by Reviewer#1, which could only be from a leading expert in this field. Yes, in our original manuscript, we did not rigorously investigate the genetic relationship between inactive mTORC1 and NHR-49/80. As Reviewer#1 said, the inactivation of mTORC1 could initiate the L1 development either by activating NHR-49/80 themselves (supported by the mammalian tissue culture data[1]), or directly activating similar key downstream target of NHR-49/80 instead.

To answer these questions, we first have performed several experiments to check whether inactive mTORC1 upregulates the NHR-49/80 activity. We found knocking down mTORC1 by *daf-15* RNAi also increased the nuclear localization of NHR-80 to an extent similar to palmitic acid supplementation (Figure S4Q, R). These data support our model that palmitic acid could activate NHR-80 by mTORC1 inactivation.

Interestingly, we found that *daf-15* RNAi could slightly further increase the nuclear localization of palmitate-treated animals (Figure S4Q, R). In addition, unlike palmitic acid supplementation, the inactivation of mTORC1 by *raga-1(lf)* could moderately increase the nuclear localization of NHR-49 (Figure S4S, T).

In addition, we performed RNA seq experiments in WT L1 animals fed with various nutrients and *raga-1* L1 animals. We found that *raga-1* and PA-supplemented animals shared partial overlap about their impact on NHR-49 downstream targets （such as *fat-7, ech-1.1, nhr-13*, and *nhr-181*）(Figure S5B).

We believe these differences were likely due to the extent of mTORC1 inactivation by *daf-15* RNAi or *raga-1(lf)* was stronger than the LCFA supplementation. Alternatively, mTORC1 likely has pleiotropic functions that only part of them was contributed by LCFA supplementation.

*TORC1 is known to activate translation. What would the authors say about translation? Does FIB-1 antibody staining data reflect the status of translation? If mTORC1 activity is still positively correlated with translation under the testing conditions, then the relationship between translation and developmental initiation may be discussed.*

In addition, we also followed reviewer#1’s question to determine whether inactive mTORC1 initiated the L1 development via downregulating the translational activity. We fed L1 *C. elegans* with cycloheximide (a eukaryotic translation inhibitor) and found it could not trigger the L1 development under starvation (Figure S2D). In the contrary, it blocked the palmitate-induced L1 development (Figure S2D). These data suggest that the low translational activity was not a major reason to initiate the development of *raga-1* animals.

Based on the data above, we modified our main text and discussion accordingly. We also added the data above in the main text.

*5. Figure 4F. The effect of over expression of NHR-80 in the intestine and hypodermis with PA addition was good, but it still just indicates that the NHR is required in at least one of the tissues for the function, and it is not the key evidence that the protein mediates the PA signal. Because the overexpression effect under the condition without PA addition was significant but much weaker, it may not be used as the clear evidence for NHR-80 being the major factor that mediates the PA signal. A good piece of supporting evidence for NHR-80 being a downstream factor of PA was the change in nuclear localization (Fig. 4D-E). The weaker effect of overexpression of NHR-80 in PA(-) condition may be due to the inability of high level expression to constitutively activate the NHR protein. If there is no better way to make a hyperactive NHR-80/49, the authors may want to modify the discussion about the results to point out the limitation/caveat of the data.*

We appreciate and totally agree with the comments from Reviewer #1. Following Reviewer#1‘s suggestion, we crossed several reported NHR-49 gf alleles (which suppress the cold-sensitive phenotype of a *paqr-2*/adiponectin receptor mutant) with the seam cell marker. We found one *gf* allele *nhr-49(et8)* had a moderate development-promotion effect without supplementation of palmitic acid under starvation, similar to the overexpression of NHR-80 (Figure S4P). However, the other two *gf* alleles *nhr-49(et7)* and *nhr-49(et13)* did not develop, even under the supplementation of palmitic acid (Figure S4P), possibly because they have different activities on their protein-binding and downstream target genes[2].

Based on these data, we followed reviewer#1’s suggestion and added several sentences in the discussion to point out that besides activating NHR-49/80, LCFA could also parallelly promote the L1 development via other pathways. We also added the NHR-49 gain-of-function data in the main text.

*6. The authors need to make additional efforts to improve the writing of the manuscript. There are some grammatical errors and awkward/confusing sentences in the manuscript. A few examples are listed below:*

*- in the writing, the authors should not use "the" in front of FEDUS.*

We appreciate the correction and modified the text accordingly.

*- Last sentence in the abstract: the semi-colon was not used correctly. Should just use period and then start a new sentence.*

We appreciate the correction and modified the text accordingly.

*- page 2, third line from bottom. "These findings suggest that...". The authors may want to consider revising the sentence to be more precise in the message. It is possible all major macronutrients are used as signals in some developmental decision, but in a given specific decision,* *only one or two were used as the signal to mark the status of overall nutrient levels.*

We appreciate the correction and modified the text to “These findings suggest that in a given specific developmental stage, only part of key macronutrients is used as the signal to indicate the overall nutrient quality and contributes to the developmental decision in animals.”.

*- page 3, line 7, the comma needs to be deleted.*

We appreciate the correction and modified the text accordingly.

*-page 3, line 10, "by simply dietarily supplementing them individually." very awkward. May be changed to: by supplementing each nutrient individually...*

We appreciate the correction and modified the text accordingly.

*- page 4, line 8, at the first larval stage (L1)*

We appreciate the correction and modified the text accordingly.

*page 4, line 4-2 from the bottom, awkward sentence.*

We appreciate the correction and modified the text to “Moreover, a classical chemotaxis experiment also indicated that only animals fed with palmitic acid showed mature and functional AWC neurons capable of responding to the chemical cue butanone.”

*page 5, line 2. may change to "We found that animals fed palmitic acid, but not glucose or AA mix, showed ...."*

We appreciate the correction and modified the text accordingly.

*page 14, line 1 under "Discussion". "A few macronutrients" could mean many. There are only a few macronutrients, at most, anyway. The authors need to rewrite the sentence to eliminate the potential confusion. May be just say one or two specific nutrients.*

We appreciate the correction and modified the text to “only one or two macronutrients could sufficiently activate the postembryonic development in *C. elegans*”

*page 15, line 1. "surprisingly intriguing" is awkward, use only one of them. Also need to add a comma after LCFA.*

We appreciate the correction and modified the text to “an intriguing finding”

*page 16, try not use the word "we guess".*

We appreciate the correction and modified the text to “We hypothesized that”.

*page 16, line 8. The last sentence of the paragraph is confusing. I would delete it. No one would understand without going to detail to explain. Not needed.*

We appreciate the correction and deleted it accordingly.

*- in general, try not use "very" too often. such as "very interesting".*

We appreciate the correction and deleted those words accordingly.

*- refence 40 seems to be the same paper of reference 84.*

We are sorry for the confusion. Ref#40 is the preprint version of Ref#84. We deleted it accordingly and cited the published paper instead.

*Rev. 2:*

*The authors describe a seemingly new nutrient checkpoint in C. elegans development that they name FEDUS. The authors report that the addition of palmitic acid alone during starvation can promote development and that this developmental checkpoint occurs earlier than the previously described DAF-16 dependent checkpoint.*

*They show that the effects of PA can be suppressed (partially) by loss of NHR-49 or NHR-80 and suggest that PA functions as a ligand to activate these transcription factors. They also show that PA treatment promoted relocalization of peroxisomes to the apical side of the intestine and that unc-116 mutation, which inhibits this relocalization, disrupts the FEDUS phenotype. The authors propose that PA promotes the release of a peroxisomal ligand that induces development by acting on chemosensory neurons.*

*The individual experiments are generally well done and controlled. The description of the FEDUS is interesting and novel. There are some interesting pieces described. However, I find some of the conclusions overstated and the overall model needs more validation to solidify links between individual observations.*

We really appreciate the positive response from Reviewer#2. We also thank the constructive suggestions raised below. We followed those suggestions and substantially improved our manuscript through additional experiments, discussion and other modifications.

*Major issues:*

*1. Altogether, the evidence that PA acts as a ligand for NHR-49/80 is not convincing.*

*The authors suggest that PA functions as a ligand for NHR-49/NHR-80. The fact that these two NHRs function together has previously been demonstrated. NHR-49 is also reported to function with NHR-66, but there is no mention as to why only NHR-80 was tested. In any case, the authors show suppression of the FEDUS phenotype when either NHR-80 or NHR-49 are absent. However, this suppression is not complete, which does not support the idea that PA functions solely by acting as a ligand for this complex. This is never discussed. In addition, with partial suppression from each mutant, it would seem logical to test the double mutant to validate the idea that they are working as a complex and not independently.*

We appreciate reviewer#2’s sophisticated questions. Following those suggestions, we tested the function of other NHR-49 partners such as NHR-66 and MDT-15 in FEDUS. We found *nhr-66* also significantly blocked FEDUS (Figure S4G), but its nuclear localization did not change under different nutrient conditions (Figure S4N, O). Meanwhile, MDT-15 RNAi knockdown did not have an obvious effect (Figure S4I) (we couldn’t obtain the *mdt-15; str-2::GFP* animals because they were potentially genetically linked).

We failed to get the *nhr-49/80* double mutant in our hand because the crosses from single mutants were not successful (*str-2::GFP* animals did not grow or mate very well under genetic mutations). And RNAi of these genes are also not very effective. Just like Reviewer#1 suggested, we found overexpression of NHR-80 or *nhr-49* gf allele *nhr-49(et8*) could only moderately promote the L1 development (Figure S4P), while palmitic acid or inactivation of mTORC1 could strongly promote the L1 development. we believe NHR-49/80 is not likely the only target of palmitic acid. Other hormone receptors such as NHR-66 could also play a role in FEDUS.

We have added these data and related analyses in the discussion.

*The authors show that NHR-80 is relocalized to the nucleus in response to PA, which does support a potential effect on NHR-80, but it does not show a direct link. PA could just as easily regulate another regulator of NHR-80.*

We followed the reviewer’s suggestion and tried to verify the direct interaction between LCFA and NHR-49/80. However, many biochemical attempts were unsuccessful since most NHR-49/80 proteins were degraded before we could carry assays such as Differential Scanning Fluorimetry or Microscale Thermophoresis.

Therefore, we adopted an HEK293 cell-based reporter assay. Indeed, we found palmitic acid, but not heptadecanoic acid (C17:0 fatty acid), could activate the human NHR-49 ortholog PPARα (Figure 4U). These data not only suggest that LCFA such as palmitic acid could be a ligand/activator of NHR-49/PPARα, it also implies that the underlying mechanism could be conserved among species.

We have added data in the main text and also acknowledged the technical limitation (no direct binding assay data) in the discussion.

*"Taking together, these data suggest that although NHR-49 and NHR-80 are both*

*required for the FEDUS, only intestinal nucleus-localized NHR-80 critically mediates the FEDUS phenotype" The meaning of "critically mediates" is unclear.*

For the last question, we are sorry about the confusion, we modified the sentence to “NHR-49, NHR-66, and NHR-80 are all required for FEDUS; and palmitic acid/inactive mTORC1 induced intestinal nucleus-localization of NHR-80 likely played a more important role in FEDUS”

We added these data in the main text.

*2. The proposed model, that relocation of peroxisomes to the apical membrane facilitates secretion of a peroxisome-derived development-promoting hormone is not well supported. The hypothesis is primarily based on corelative data. How do the authors know that the observed relocalization is a primary response and not secondary to the initiation of growth? For that matter, what is the evidence that the signal for FEDUS comes from the intestine?*

*The requirement for UNC-116 doesn't provide a great deal of support. UNC-116 functions in many different cell types to aid in the localization of many different structures. If neuronal signaling is needed, how can the authors be sure it isn't functioning there? UNC-116 is also required for the transport of dense core vesicles. Given the requirement for the dense core vesicle binding protein UNC-31 in FEDUS, it seems reasonable to expect that loss of UNC-116 would have the same effect as loss of UNC-31 (loss of either would prevent the release of dense core vesicles). As such, there is not a strong link between the peroxisomal localization and causal effects.*

We appreciate the concerns from Reviewe#2. To address these concerns, we overexpressed *unc-116* in a tissue-specific manner and found intestinal but neuronal expression of *unc-116* could restore the FEDUS in *unc-116(-)* (Figure S4D). These data support the localization of *unc-116* mainly functions in the intestine to mediate the signal of LCFA. We believe that the relatively minor effect of *unc-116(-)* in neurons was possible due to the *unc-116* mutant we used was a hypomorph allele *unc-116(e2310),* and it did not affect DCV run velocities but only affected the general movement pattern of DCVs [3]. The strong loss-of-function *unc-116* is lethal [4]. Therefore, the DCV transportation could only be partially affected in *unc-116(e2310*), instead of being fully blocked by the *unc-31* deletion allele *unc-31(e928).*

*3. Statistical analysis. In the methods section, it is stated that all experiments, unless noted, were analyzed with a t-test. However, many figures include multiple comparisons, for which a t-test would not be appropriate. Further, there is no mention of* *a correction for multiple hypothesis testing in any of these figures.*

We appreciate the valuable suggestions and modified the statistical analysis accordingly. We also added the multiple hypothesis testing correction in the method.

*4. The discussion was somewhat unsatisfying. The authors should describe the model in more detail in the discussion. The data with daf-16 are interesting - what does the enhancement with daf-16 mutation mean? Does the addition of PA allow daf-16 mutants to progress through development more rapidly? What does this mean in terms of daf-16 mutants bypassing arrest? How do daf-16 mutants pass the first PA-dependent checkpoint during starvation?*

We appreciate the suggestions from Reviewer#2 (such a question has also been raised by Reviewer#1). We added several sentences in the first paragraph to describe the model in detail. In addition, we also discussed the dual roles of mTORC1 in early L1 development (in the discussion). Moreover, we followed reviewe#2’s suggestion to discuss the role of *daf-16*. The *daf-16* mutant animal itself cannot bypass the PA-dependent first checkpoint (Figure S5K), suggesting activation of IIS is not sufficient to overcome the first diapause. But when PA exists, the *daf-16(-)* animals grew better and their development advanced further (Figure S5K). Such data agree with previous findings that *daf-16(-)* could bypass a later L1 checkpoint guarded by ethanol and amino acids. Taken together, these data suggest that complete L1 development has two sequential checkpoints. The first checkpoint is initiated by LCFA and low mTORC1 activity, and the second checkpoint is controlled by amino acid and high/IIS-mTORC1 activity.

We add them to the discussion.

*Minor issues*

*The meanings of statistical indicators (***) are not present in all figure legends; similarly**, the statistical test used is often missing from the figure legend.*

We appreciate the valuable suggestions and modified the statistical indicators and added the test in figure legends accordingly.

*In the last paragraph of the discussion, the authors discuss the idea of nutritional checkpoints as a novel idea proposed here, but this idea has been proposed by others (and in C. elegans) - these should be referenced.*

We appreciate reviewer#2’s suggestion and added related references in the large paragraph. In addition, to avoid misleading, we significantly modified the last paragraph and described more detail about what’s known and what’s new in this work related to the nutrient checkpoint theory.

*Methods, strains and transgenes should be properly named (with transgene names and strain names).*

We appreciate reviewer#2’s suggestion and modified the name accordingly.

*Many of the strains used should be referenced with the papers they were originally described in.*

We appreciate reviewer#2’s suggestion and added the reference accordingly.

*Authors note that population density can have an effect on FEDUS, the density of arrested L1s used for experiments should be noted in the methods section*

We appreciate reviewer#2’s suggestion and added the density (3-4 animals/µl) in the method accordingly.

*Figure 1F - typo in the panel title*

We appreciate reviewer#2’s suggestion and modified it accordingly.

*Figure 1M - what are the triangles? Please define in legend.*

We are sorry for the confusion. The triangles indicated the mean of three replicas. We have removed them in the figure accordingly.

References

1.	Venn-Watson, S., R. Lumpkin, and E.A. Dennis, *Efficacy of dietary odd-chain saturated fatty acid pentadecanoic acid parallels broad associated health benefits in humans: could it be essential?* Scientific Reports, 2020. **10**(1): p. 8161.

2.	Lee, K., et al., *Gain-of-Function Alleles in Caenorhabditis elegans Nuclear Hormone Receptor nhr-49 Are Functionally Distinct.* PLoS One, 2016. **11**(9): p. e0162708.

3.	Laurent, P., et al., *Genetic dissection of neuropeptide cell biology at high and low activity in a defined sensory neuron.* 2018. **115**(29): p. E6890-E6899.

4.	Drozd, C.J. and C.C. Quinn, *UNC-116 and UNC-16 function with the NEKL-3 kinase to promote axon targeting.* Development, 2023. **150**(18).
