# Supplementary material for: Free long-chain fatty acids trigger early postembryonic development in starved Caenorhabditis elegans by suppressing mTORC1
Source: PLoS Biol. 2024 Oct 22;22(10):e3002841. doi: 10.1371/journal.pbio.3002841 (PMC11530034; doi:10.1371/journal.pbio.3002841)
Supplement: Attachment — Submitted filename: Response letter to Reviewers.docx [file pbio.3002841.s014.docx]

*Reviewers' comments*

*Rev. 1:*

*The authors have an impressive job in address all questions from both reviewers. I am very satisfied with their strong effort. The technical aspects of the manuscript has been further improved and the high significance of the work is solidified. The acceptance by PLoS Biology is highly recommended.*

*The authors have modified title based on my suggestion on the original manuscript. I think the new title is not good as it may mean that the TOR role was the major/only finding in the paper. In addition, "under starvation" may be at the end for the new title. Here are the two alternative titles I would suggest:*

*1. Modified new title to: "Free long chain fatty acid solitarily launches early postembryonic development in starved Caenorhabditis elegans and does so by suppressing mTORC1". The only potential weakness is that it may be too long if PLOS Biology has a length restriction.*

*2. Go back to the original title, without mentioning the role of TOR. It lacks the mechanistic message as I complained in the original review, but at least it would make it clear regarding the most novel aspect of the finding (FA role).*

Response

We really appreciated the comments and suggestions from Reviewer#1. We also modified the title to “*Free long chain fatty acids trigger early postembryonic development in starved Caenorhabditis elegans by suppressing mTORC1*” .

*Rev. 2:*

*I thank the authors for the inclusion of additional data that supports some of the findings. I find the manuscript much improved.*

We really appreciated the comments and suggestions from Reviewer#2.

*The manuscript text will need a thorough re-write as it still contains many typographical and grammatical errors. In some places, it is also difficult to read (specifically the methods). For example: "Eggs laid naturally by well-fed str-2::gfp C. elegans and L1 C. elegans on NGM plates were collected for growing to adult and subsequent bleaching. Noted that egg/L1 should be adjusted at a suitable number/plate to avoid food deprivation."*

We thank reviewer#2’s suggestion and have made another effort to improve the language, especially in the method session.

*Methods should indicate the source of the fatty acids used (I'm not sure if this is in supplemental Table 2 - I was not able to open it).*

We thank reviewer#2’s suggestion and related information is listed in the supplemental table S2.

*The discussion regarding daf-16 is still a little unsatisfying; it seems a bit at odds with findings from the Baugh lab (and others). The authors note in the discussion "Such a model could also explain that daf-16(-) itself cannot initiate the first checkpoint", this is true in the conditions used in this manuscript (in M9 buffer) However, given the FEDUS check point is described as preceding the daf-16 checkpoint, it fails to explain why daf-16 mutants starved in L1 (in S-basal) show development of cells in the M and V lineages (Baugh and Sternberg, Current Biology 2006). A hypothesis is needed to explain how daf-16 mutants in S-basal are bypassing the FEDUS checkpoint. This can be addressed in the text without further experiments.*

We thank Reviewer #2 mentioned the Baugh and Sternberg (2006, Ref#90) study. In that work, *daf-16* mutant could bypass the early L1 arrest in S basal, which Reviewer2 suggested that it seems inconsistent with our results. However, this discrepancy has been addressed by Fukuyama et al. (2015, Ref#21). They found that the S-basal buffer used by the Baugh and Sternberg (2006) study contains approximately 0.1% (~22mM) ethanol (used to dissolve cholesterol), which was necessary for the *daf-16* mutant to cause L1 development. In contrast, our study utilized an isotonic M9 buffer that does not contain any ethanol (we mentioned that in the beginning of our result part “We took the advantage of a commonly used method to synchronize newly hatched C. elegans at the first larval stage (L1) by suspending them in the M9 buffer, a saline-like medium without any organic nutrients such as ethanol”). We further clarified this in the discussion, as suggested by Reviewer #2.
